# The dynamic adrenal response of children to cardiac surgery and cardiac catheterization

**DOI:** 10.1210/clinem/dgag206

**Published:** 2026-05-13

**Authors:** Daniel Paul Fudulu, Isabella Marinelli, Daniel Galvis, Eder Zavala, Terrie Walker-Smith, Jonathan Evans, Fanoula Papadopoulou, Thomas Upton, Aravind Ramesh, Filippo Rapetto, Yvonne Kershaw, Jan Fox, Angela Aramburo, Serban Constantin Stoica, Massimo Caputo, Andrew Parry, Duncan Macrae, Guido Michielon, Johann Andreas Hoschtitzky, Karen Sheehan, Martijn van Faassen, Ido Kema, Gianni Davide Angelini, Stafford Lightman, Ben Gibbison

**Affiliations:** Bristol Medical School, University of Bristol, Bristol BS8 1TH, UK; Department of Cardiac Surgery, University Hospitals Bristol and Weston NHS Foundation Trust, Bristol BS2 8HW, UK; Department of Mathematics and Statistics, University of Exeter, Exeter EX4 4QJ, UK; National Grid (Avonbank), Bristol BS2 0TB, UK; School of Mathematics, University of Birmingham, Birmingham B15 2TT, UK; Centre for Biological Timing; Division of Diabetes, Endocrinology and Gastroenterology, Faculty of Biology, Medicine and Health, University of Manchester, Manchester M13 9PL, UK; Bristol Trials Centre, University of Bristol, Bristol BS8 2BN, UK; Bristol Trials Centre, University of Bristol, Bristol BS8 2BN, UK; Department of Cardiac Surgery, University Hospitals Bristol and Weston NHS Foundation Trust, Bristol BS2 8HW, UK; Bristol Medical School, University of Bristol, Bristol BS8 1TH, UK; Bristol Medical School, University of Bristol, Bristol BS8 1TH, UK; Department of Cardiac Surgery, IRCCS Ospedale Policlinico San Martino, Genova 16132, Italy; Bristol Medical School, University of Bristol, Bristol BS8 1TH, UK; Bristol Medical School, University of Bristol, Bristol BS8 1TH, UK; Department of Paediatric Intensive Care, Royal Brompton and Harefield NHS Foundation Trust, London SW3 6NP, UK; Bristol Medical School, University of Bristol, Bristol BS8 1TH, UK; Department of Cardiac Surgery, University Hospitals Bristol and Weston NHS Foundation Trust, Bristol BS2 8HW, UK; Bristol Medical School, University of Bristol, Bristol BS8 1TH, UK; Department of Cardiac Surgery, University Hospitals Bristol and Weston NHS Foundation Trust, Bristol BS2 8HW, UK; Bristol Medical School, University of Bristol, Bristol BS8 1TH, UK; Department of Cardiac Surgery, University Hospitals Bristol and Weston NHS Foundation Trust, Bristol BS2 8HW, UK; Department of Paediatric Intensive Care, Royal Brompton and Harefield NHS Foundation Trust, London SW3 6NP, UK; Congenital Heart Surgery & Heart Department, IRCSS Gaslini Children's Hospital, Genova 16147, Italy; Department of Paediatric Intensive Care, Royal Brompton and Harefield NHS Foundation Trust, London SW3 6NP, UK; Department of Cardiac Surgery, University Hospitals Bristol and Weston NHS Foundation Trust, Bristol BS2 8HW, UK; Department of Laboratory Medicine, University of Groningen, 9713 GZ Groningen, Netherlands; Department of Laboratory Medicine, University of Groningen, 9713 GZ Groningen, Netherlands; Bristol Medical School, University of Bristol, Bristol BS8 1TH, UK; Department of Cardiac Surgery, University Hospitals Bristol and Weston NHS Foundation Trust, Bristol BS2 8HW, UK; Bristol Medical School, University of Bristol, Bristol BS8 1TH, UK; Bristol Medical School, University of Bristol, Bristol BS8 1TH, UK; Department of Cardiac Surgery, University Hospitals Bristol and Weston NHS Foundation Trust, Bristol BS2 8HW, UK

**Keywords:** HPA axis, pediatric cardiac surgery, glucocorticoid dynamics, dynamic markers, mathematical modeling

## Abstract

**Context:**

The hypothalamic–pituitary–adrenal (HPA) axis is the key homeostatic system regulating the response to surgical stress. Imbalances in HPA axis hormones increase morbidity and mortality in children after cardiac surgery. Despite this, the physiology of the HPA axis in children undergoing cardiac surgery is poorly understood, leading to controversies in clinical practice.

**Objective:**

To characterize dynamic HPA axis responses in children undergoing cardiac surgery and to determine age- and procedure-related differences in cortisol and cortisone physiology.

**Methods:**

We recruited children (0-18 years) undergoing cardiac surgery with cardiopulmonary bypass or cardiac catheterization. Tissue-free cortisol and cortisone were sampled every 20 minutes for up to 24 hours via microdialysis, alongside serum adrenocorticotropic hormone (ACTH), cortisol, cortisol-binding globulin (CBG), and inflammatory markers. We developed dynamic markers to quantify age- and procedure-dependent differences in hormonal responses and built a mathematical model to explain them.

**Results:**

Neonates undergoing surgery showed higher free cortisol and cortisone AUC and peak concentrations than catheterization patients. Neonates had higher peaks of cortisol and cortisone than older children undergoing surgery. The much higher tissue cortisone levels observed in neonates can be explained by enzymatic interconversion between cortisol and cortisone, likely due to persistent fetal high 11-βHSD2 activity and reduced 11-βHSD1 activity. Low postoperative blood cortisol and CBG values in neonates resulted in high free cortisol peaks in interstitial fluid during and after surgery.

**Conclusion:**

Neonates differ physiologically, with higher free cortisol levels that more readily diffuse into interstitial tissues, with implications for perioperative management.

Around 1 in 100 babies are born with congenital heart defects. Seventy percent of these will require either surgery or percutaneous intervention in the first year of life, and many require repeated interventions (1). Despite a substantial improvement in outcomes since the start of the 21st century (2), there are still considerable morbidity and mortality risks associated with cardiac surgery. A key contributor is the acute, systemic inflammation triggered by tissue damage and cardiopulmonary bypass (CPB). In contrast, acute systemic inflammation is rarely seen in patients undergoing percutaneous procedures.

Cortisol, produced by activation of the hypothalamic–pituitary–adrenal (HPA) axis, is released in response to stress, and also functions to suppress inflammation. Deficiency of cortisol is associated with uncontrolled inflammation and cardiovascular collapse. Therefore, supranormal doses of exogenous corticosteroids are frequently given to children undergoing cardiac surgery, both to correct perceived insufficiency and to harness some of the anti-inflammatory effects (3). However, there is little evidence for the effectiveness of this strategy in a mixed pediatric cardiac surgical population (4-9).

The HPA axis is well characterized in adults. Underlying the circadian rhythm of cortisol, is an *ultradian* rhythm of pulsatile release of cortisol from the adrenal gland. Typically, high amplitude pulses are seen in the morning while smaller amplitude pulses are observed in the evening and early nighttime (10). These pulses continue to be observed after cardiac surgery in adults with greatly increased peaks of cortisol (11). Cortisol rhythms encode information beyond that of the absolute level of cortisol and are therefore important for signaling (12, 13). In addition to the central, HPA axis regulation, cortisol availability during stress is regulated at the tissue level by the 11 beta-hydroxysteroid dehydrogenases 1 and 2 enzymes that interconvert active cortisol to inactive cortisone (14-16)

In children, the dynamic physiology of the HPA axis is less well understood. A major challenge in studying the dynamics of the HPA response to surgery in neonates and small children is that their small blood volume prevents taking multiple blood samples without harm. We overcame this by using a high-frequency, automated microdialysis system that can repeatedly sample free cortisol and cortisone from interstitial fluid (17, 18). Using this technology, we assessed the previously unknown dynamic cortisol and cortisone responses to cardiac surgery and cardiac catheterization in neonates, infants, children, and teenagers. This study aims to characterize the heterogeneity of dynamic HPA axis responses to cardiac surgery and catheterism in children, develop digital biomarkers to quantify the variability in these dynamic responses, and postulate the mechanisms underpinning corticosteroid dynamics under these interventions across age groups.

## Material and methods

### Study design, participants, and variables

The study was approved by Southwest—Central Bristol Research Ethics Committee, reference 16/SW/0186. Patients were recruited between July 2017 and December 2021 in 2 UK pediatric centers. Detailed methods have been published previously (19, 20).

This was a prospective observational cohort study that aimed to recruit 48 cardiac surgery and 30 cardiac catheter patients. In the cardiac surgery cohort, we aimed to recruit 12 patients aged 0-30 days (neonates), 12 patients aged >30 days to 1 year (infants), 6 patients aged 1-5 years (children), and 18 patients aged 10-16 years (teenagers). In the cardiac catheter group, we aimed to include 6 infants, 6 children, and 18 teenagers. Based on a retrospective analysis of the number of interventional procedures performed in the previous years, we did not recruit neonates undergoing catheterization because the number of procedures in this age group was very low and projected recruitment would have been very poor.

A formal sample size calculation was not performed due to the exploratory nature of the study and the absence of prior data on this specific population and outcomes. As this represents a novel study, there were no reliable estimates of effect size or variability on which to base a calculation.

When making comparisons between age groups in the analysis, we merged the infant and children groups to form a single cohort (aged >30 days to 5 years) to mitigate against small sample sizes in each group.

Inclusion criteria: children aged <18 years, weight above 2 kg, undergoing either cardiac surgery with the use of cardiopulmonary bypass (CPB) or cardiac catheter procedures performed under general anesthesia. Exclusion criteria: recent glucocorticoid use (within 3 months of procedure), emergency procedures, thyroid disease, or disorders of the HPA axis.

### Sampling of free hormones in subcutaneous tissue

We used a validated, automated microdialysis system (17) to measure free cortisol and cortisone in subcutaneous tissue. This system was further refined to enable sampling reliability in pediatric patients (20). A 20 kDa cutoff linear microdialysis catheter (membrane length 30 mm, mDialysis, Sweden) was inserted in subcutaneous tissue of the left lower abdominal quadrant immediately after induction of anesthesia and any placement of central venous and arterial catheters. Subcutaneous microdialysate sample collection occurred every 20 minutes for up to 24 hours.

### Blood sampling

Blood samples were taken throughout the study period from *in situ* vascular catheters.

#### Cardiac surgery cohort

Serum total cortisol, adrenocorticotrophic hormone (ACTH), interleukin 1 (IL-1), interleukin 4 (IL-4), interleukin 6 (IL-6), interleukin 8 (IL8), interleukin 10 (IL10), and tumor necrosis factor-alpha (TNF-α) were measured at 7 time points for infants and older children (preoperative assessment, before anesthesia induction, pre-CPB, post-CPB, and at 6, 12, and 24 hours postoperatively) and at 6 time points for neonates (as above, but with no post-CPB time point). Cortisol-binding globulin (CBG) was measured at preoperative assessment, anesthesia induction, 6 hours post-CPB and 24 hours post-CPB.

#### Cardiac catheter group

Due to the shorter intervention time and elapsed time under general anesthesia, the serum cortisol, ACTH, CBG, cytokines, and steroid hormones were measured after induction of anesthesia only.

### Assays

Cortisol and cortisone were quantified in the microdialysate by isotope dilution liquid chromatography-tandem mass spectrometry (LC-MS/MS) (21).

Samples for ACTH were collected in chilled 2 mL EDTA tubes and stored on ice for a maximum of 60 minutes before centrifugation at 4 °C and then stored at −80 °C until assay. Total blood cortisol and ACTH were measured by solid phase, chemo-luminescent enzyme linked immunoassay (ECLIA) using the Cobas e602 modular analyzer (Roche Diagnostics Ltd, West Sussex, UK). Inflammatory mediators were assayed using the Luminex® Multiplex system (Thermo Fisher Scientific—Waltham. MA). Cortisol-binding globulin was assayed using Iodine radioimmunoassay (DiaSource, Louvain-La-Neuve, Belgium).

### Dynamic markers

To quantify changes in hormonal profiles between groups, we identified 4 mathematical dynamic markers (DMs) (18): (1) area under the curve (AUC)—the total amount of hormone secreted over time, normalized to the duration of sampling; (2) secretion peak—the maximum hormone concentration detected within each sampling period; (3) hormonal peak density—the ratio between the secretion peak and the AUC; (4) prominence—a measure of a peak's differentiation from its neighbors based on its height and location relative to other peaks.

The 4 DMs encapsulate information about the total amount of secreted hormone (AUC), as well as the shape of the hormone profile (peak density and prominence) and its peak (secretion peak). The normalized cortisol ratio, calculated as F/(E + F), where F is the cortisol concentration and E is the cortisone concentration (18), indicates the fraction of active vs total glucocorticoid available.

### Mathematical modeling

We built a mathematical model to gain insights into the physiological processes underlying dynamic changes in hormonal patterns. These processes are represented by model parameters. Fitting the mathematical model to microdialysis time series data allowed us to estimate the parameter values across participants. Cortisol and cortisone were modeled such that they may be interconverted between each other by corresponding subtypes of the 11β-HSD enzymes. The model represents enzymatic interconversion as Michaelis–Menten type kinetics and hormone turnover as first-order degradation. This approach allows for the relevant physiological processes determining glucocorticoid concentrations to be accounted for simultaneously. Therefore, the model mimics a dynamic, continuously changing system as seen in vivo, rather than a static in vitro system. Full details of the model and its calibration are included in Supplementary Material (22). The governing equation for the model is:


$$\frac{\mathrm{dE}}{\mathrm{dt}}=V_{F}\frac{F}{K_{F}+F}-V_{E}\frac{E}{K_{E}+E}-\gamma E$$

where F and E are the concentrations of cortisol and cortisone, respectively. K_F_ and K_E_ are Michaelis–Menten constants that measure the 11β-HSD enzymatic affinity for cortisol and cortisone, respectively. V_F_ and V_E_ are the maximum velocities of the reactions catalyzed by 11β-HSD enzymes that produce cortisol and cortisone, respectively. *γ* is the rate of hormone removal, assumed the same for cortisol and cortisone.

The model was fitted to microdialysis data from patients, which allowed estimation of parameter values representing the processes contributing to observed levels of cortisol and cortisone.

### Computational and statistical analysis

Multigroup comparisons of serum analytes and dynamic markers were performed using the Kruskal–Wallis nonparametric test with Bonferroni post hoc correction for multiple comparisons. Computational simulations of the model and statistical analysis used MATLAB v.2022a (The MathWorks, Natick, MA) and R software (RStudio 2023.12.1 + 402 “Ocean Storm” Release for macOS) packages (gtsummary).

## Results

### Recruitment and cortisol sampling

Hormonal profiles were collected from 52 participants. In the cardiac surgery group, we recruited 6/6 neonates, 12/12 infants, 6/12 children, and 11/18 teenagers. In the cardiac catheter group, we recruited 3/6 infants, 1/6 children, and 13/18 teenagers. Reasons for recruitment failure were low numbers of patients receiving interventions in a particular age cohort, or family/nursing concerns over maintaining the microdialysis system placement in mobile younger children (age 1-5 years). No children were recruited between the ages of 6 and 10 years in either group. Thirty-five of 52 (65%) participants reached at least 8 hours of microdialysis sampling. The mean sampling durations were 20 hours (SD = 4.78 hours) for the cardiac surgery group and 14 hours (SD = 5.83 hours) for the cardiac catheter group. Reasons for early discontinuation of sampling were accidental microdialysis catheter disconnection, perioperative glucocorticoid administration, or patient request.

### Clinical characteristics and outcomes

The baseline characteristics of the participants that underwent cardiac surgery and cardiac catheterization are detailed in Table 1.

**Table 1:** Clinical characteristics of the cardiac surgery and catheter groups

Cardiac surgery group (SUR)				
	Neonates (N = 6)	Infants (N = 12)	Children (N = 6)	Teenagers (N = 11)
Age	12 [10-12] days	134 [117-169] days	1.6 (1.2-2.9) years	14.5 (14.1-15.3) years
Females, n (%)	3 (50)	4 (33)	3 (50)	4 (36)
Weight	4 [4-4] kg	5 [5-6] kg	10 [9-11] kg	47 [44-57] kg
CPB time	157 [92-203] min	123 [73-166] min	135 [106-162] min	68 [60-126] min
Aortic cross clamp time	86 [58-121] min	76 [39-92] min	52 [29-83] min	48 [37-91] min
Duration of postop ventilation	136 [123-153] hours	30 [19-96] hours	33 [25-47] hours	11 [4-22] hours
Total length of hospital stay	16 [12-24] days	9 [6-17] days	9 [6-28] days	6 [5-9] days

Cardiac catheter group (CAT)			
	Infants (N = 3)	Children (N = 1)	Teenagers (N = 13)
Age	193 [135-196] days	5 years	14.1 (13.3-15.7) years
Females, n (%)	1 (33)	1 (100)	6 (46)
Weight	6 [5-8] kg	13 [13-13] kg	59 [51-69] kg
Procedure time	73 [72-74] min	39 [39-39] min	150 [106-173] min

Values reported are median [IQR]. CPB, cardiopulmonary bypass.

There were no in-hospital deaths, no postoperative strokes, and no need for postoperative ECMO. In the cardiac surgical group, there was 1 reoperation in the neonate, 2 in the infant and 1 in the children group. All cardiac catheter patients were discharged on the day of admission.

### Microdialysis data

Figure 1A-1G shows an example of one patient's microdialysis data in each age group undergoing cardiac surgery and cardiac catheterization with general anesthesia. Subcutaneous cortisol microdialysis trends were broadly in line with blood cortisol trends.

**Figure 1:**
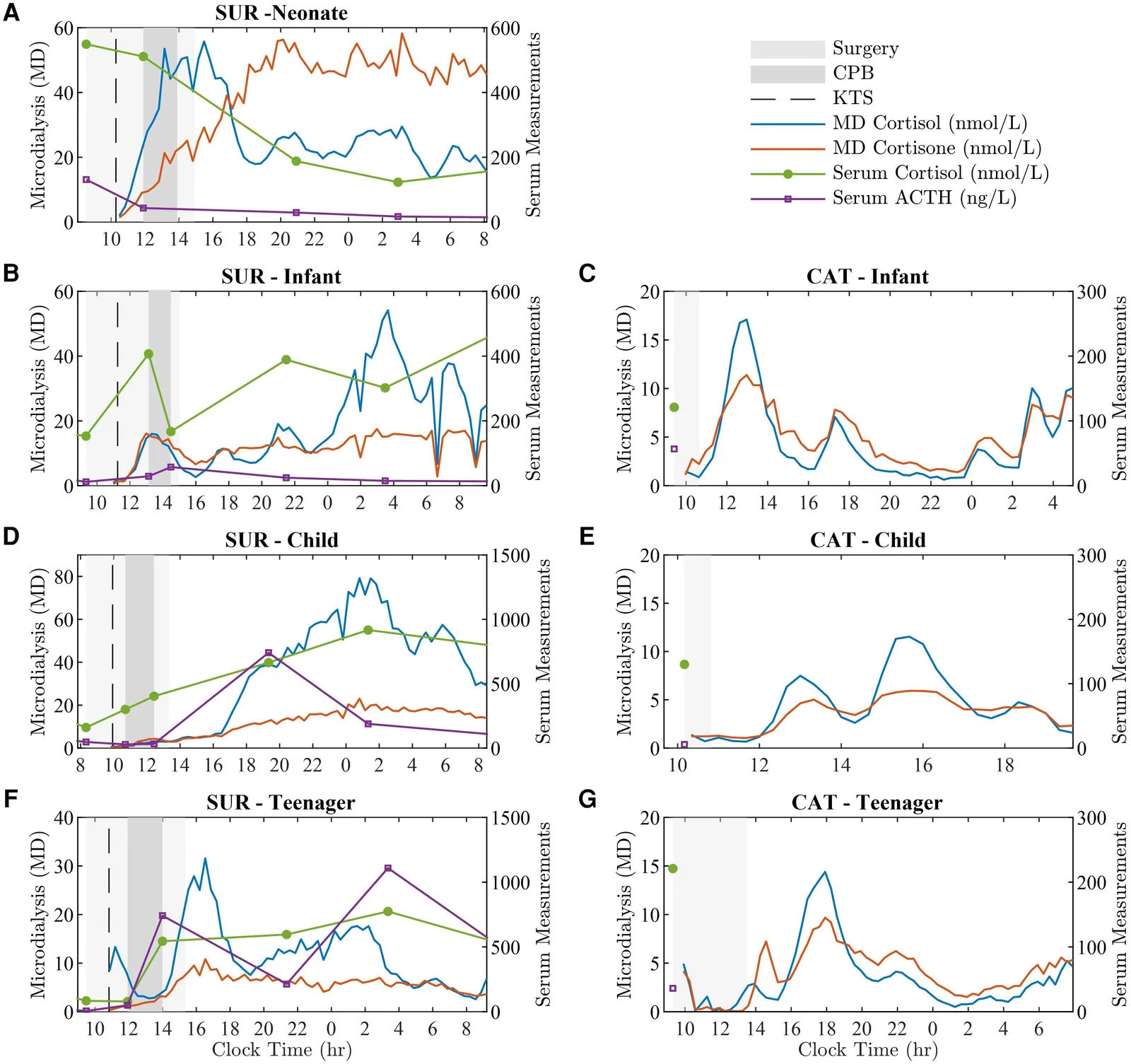
Hormonal dynamics in young patients. Examples of dynamic hormone profiles from 7 patients: neonate (A), infants (B-C), children (D-E), and teenagers (F-G) undergoing surgery (left) or catheterization (right). Contemporaneous serum and microdialysis measurements are shown. Abbreviations: CAT, catheter; SUR, surgery; CPB, cardiopulmonary bypass; KTS, knife to skin.

### Blood data

Figure 2 shows the blood data for cortisol, ACTH and the inflammatory mediators in children having cardiac surgery. Serum total cortisol was lower in neonates (159.03 ± 108.18 nmol/L at 6 hours, 223.48 ± 95.68 nmol/L at 12 hours and 402.94 ± 200.71 nmol/L at 24 hours) than the combined infants + children group (747.93 ± 437.11 nmol/L at 6 hours, 875.34 ± 348.98 nmol/L at 12 hours and 730.60 ± 290.97 nmol/L at 24 hours) at all postoperative time-points (significant at 6 hours *P* = .003 and 12 hours *P* = .001).

**Figure 2:**
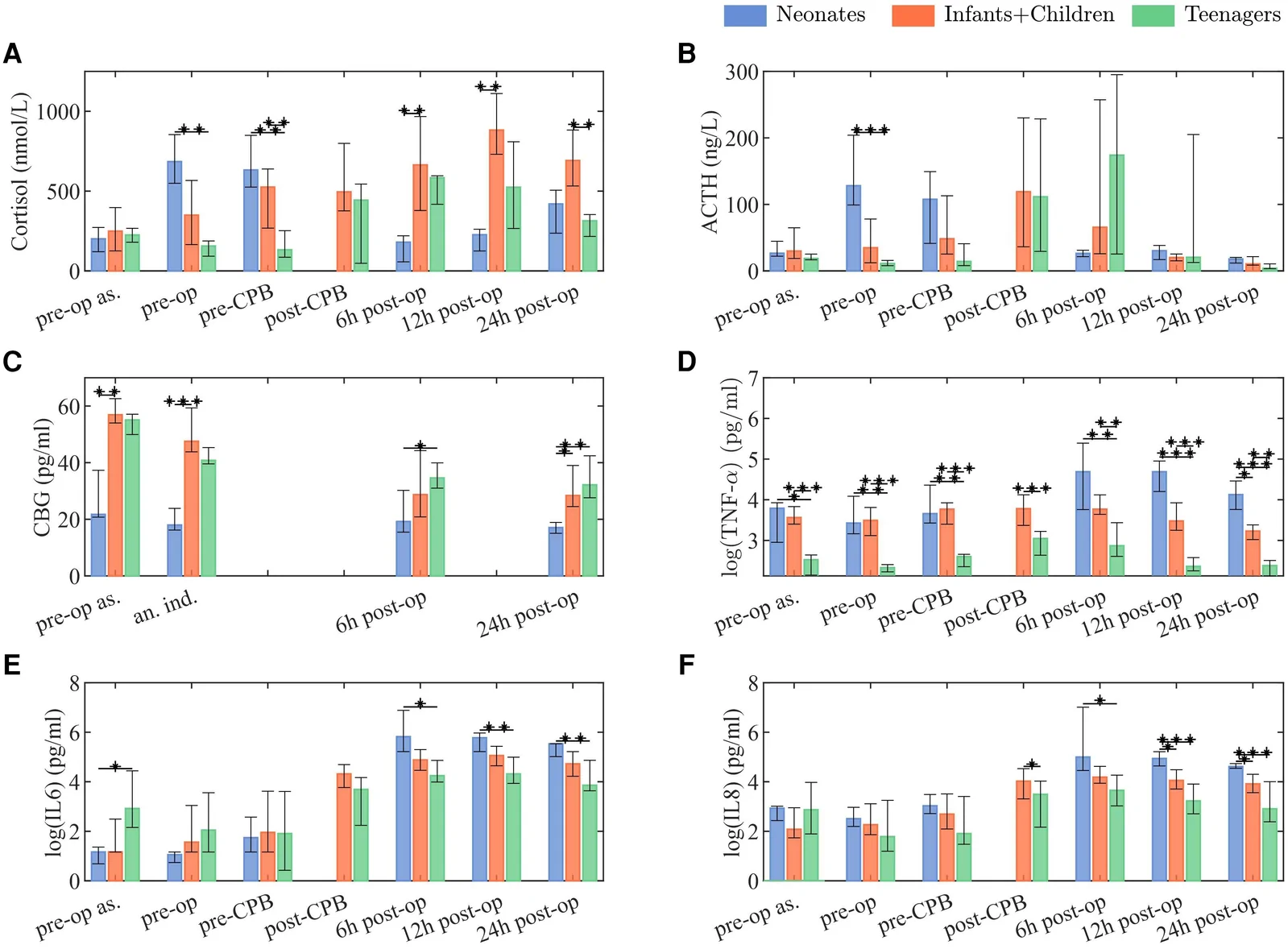
Analytes in serum from surgery patients. Serum concentrations of cortisol (A), ACTH (B), CBG (C), and log-concentrations of inflammatory markers (D-F) in young patients undergoing cardiac surgery. Blue: neonates, orange: infants + children, green: teenagers. Statistical analysis was performed employing Kruskal–Wallis nonparametric test and the post hoc Bonferroni's Test. * = *P* < .05; ** = *P* < .01; *** = *P* < .001. Abbreviations: preop as.: preoperative assessment sample; preop: before anesthesia induction; pre-CPB: before going on CPB; post-CPB: after wean from CPB; an. ind.: anesthesia induction; 6 hours postop—6 hours after end of surgery, 12 hours postop—12 hours after end of surgery, 24 hours postop—24 hours after end of surgery.

Cortisol-binding globulin was lower in neonates at all time-points (28.23 ± 10.97 pg/mL at before anesthesia induction [preop], 21.02 ± 8.16 pg/mL at anesthesia induction [an. ind.], 22.08 ± 7.75 pg/mL at 6 hours, 17.96 ± 5.87 pg/mL at 24 hours) than the combined infants + children group (67.77 ± 36.92 pg/mL at preop, 51.13 ± 11.65 pg/mL at an. ind., 31.64 ± 15.83 pg/mL at 6 hours, 30.85 ± 11.42 pg/mL at 24 hours) (significant at preop as. *P* = .001, an. ind. *P* = .0001, 24 hours *P* = .037). The CBG concentration remained constant (all *P*-values <.05) across the time in all classes.

The concentration of TNFα was lower in teenagers than the other 2 classes at all time points (all *P*-values <.05). The concentration of IL6 was higher in neonates than other age groups at all postoperative time-points (significant—*P*-values <.05—only between neonates and teenager). The concentration of IL8 was higher in neonates than other age groups at all postoperative time-points (all *P*-values <.05, except between neonates and infants + children at 6 hours *P* = .30). The concentrations of IL10, IL1β, and IL4 are shown in Fig. S1.

Children undergoing cardiac catheterization had similar values of cortisol, ACTH, CBG, and inflammatory mediators in the preoperative period as teenagers (Fig. S2).

### Dynamic markers

Patients undergoing surgery had a higher *AUC* and peaks of cortisol, cortisone and normalized glucocorticoid ratio F/(F + E), but lower density and prominence than those undergoing cardiac catheterization (Fig. S3).

Neonates had a similar AUC of cortisol, but a higher AUC of cortisone when compared to other age groups having the same procedure (surgery) (Fig. 3). They also had a similar peak of cortisol, but a higher peak of cortisone when compared to other age groups having the same procedure. The density of both cortisol and cortisone and prominence of cortisone was similar to other age groups having the same procedure type.

**Figure 3:**
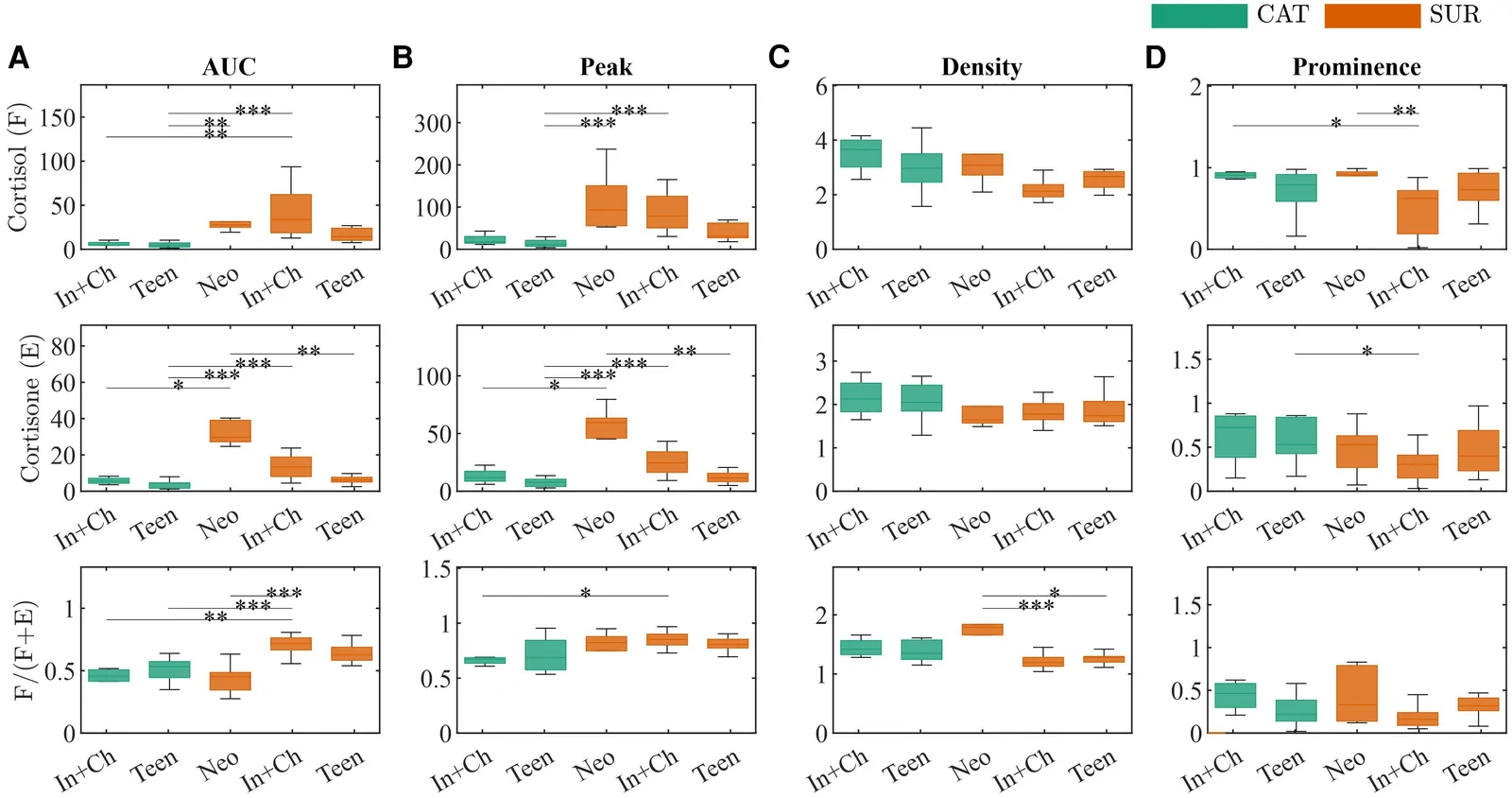
Dynamic markers grouped by age and intervention. Distributions of DM values for cortisol (F), cortisone (E) and normalized glucocorticoid ratio F/(F + E). The population is split into 3 ages and 2 types of intervention: cardiac catheterization (CAT) in green and cardiac surgery (SUR) in orange. The dynamic markers are area under the curve (AUC) (A), secretion peak (B), secretion density (C), and prominence (D). Neo = Neonates, In + Ch = Infants + Children, Teen = Teenager. Statistical analysis was performed employing Kruskal–Wallis nonparametric test and the post hoc Bonferroni's test. * = *P* < .05; ** = *P* < .01; *** = *P* < .001.

### Mathematical modeling

We modeled cortisol and cortisone enzymatic interconversion as an open-loop, parsimonious, ordinary differential equation with cortisol (F) as the input and cortisone (E) as the output (Fig. 4A). We assumed Michaelis–Menten (MM) kinetics for the enzymatic reactions, where V_max_ is the maximum reaction rate and K is the MM half-maximum constant (Fig. 4B). These parameters account respectively for the maximum enzymatic activity at substrate saturation (which can occur during the stress response) and the enzymatic affinity for its substrate (Fig. 4C). We included a pair of parameters (V_max_, K) for both directions of hormone interconversion (Fig. 4A) governed by the enzymes 11β-HSD1 (V_E_, K_E_), and 11β-HSD2 (V_F_, K_F_). We hypothesized that the variability in hormonal dynamic responses observed in this study may arise from individual differences in these parameter values (and the cortisone turnover rate γ). By fitting the model to cortisone time series data while using cortisol as the input (Fig. 4D, Fig. S4), we determined the parameter values that best fit the model to each patient's data (Fig. 5, Fig. S4). By repeating this process for all patients, we were able to find the parameter values leading to the model best fits for the entire cohort (Figs. S4 and S5), with model fits to the catheterization group (R^2^ = 0.89 ± 0.09) marginally better than to the surgery group (R^2^ = 0.85 ± 0.11).

**Figure 4:**
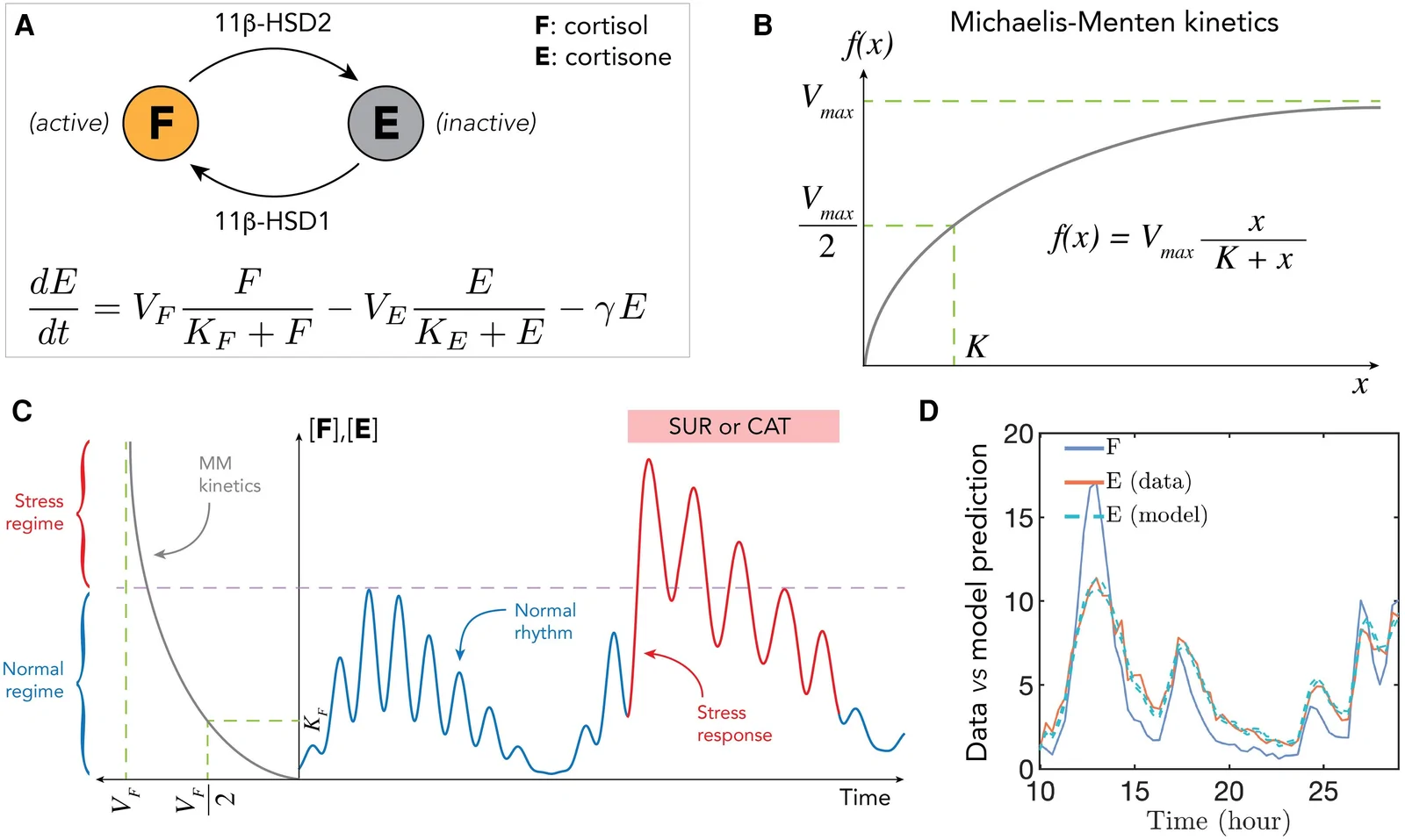
Mathematical modeling. (A) Mathematical and graphical representation of the model. (B) Simplest form of Michaelis–Menten nonlinear kinetics for enzymatic reactions. (C) Cortisol and cortisone rhythms that typically operate in the normal Michaelis–Menten regime may lead to substrate saturation during the stress response to surgery (SUR) or catheterization (CAT). (D) Hormonal profiles of cortisol (F) and cortisone (E data) for one patient alongside the confidence interval of model fits to the data (E model).

**Figure 5:**
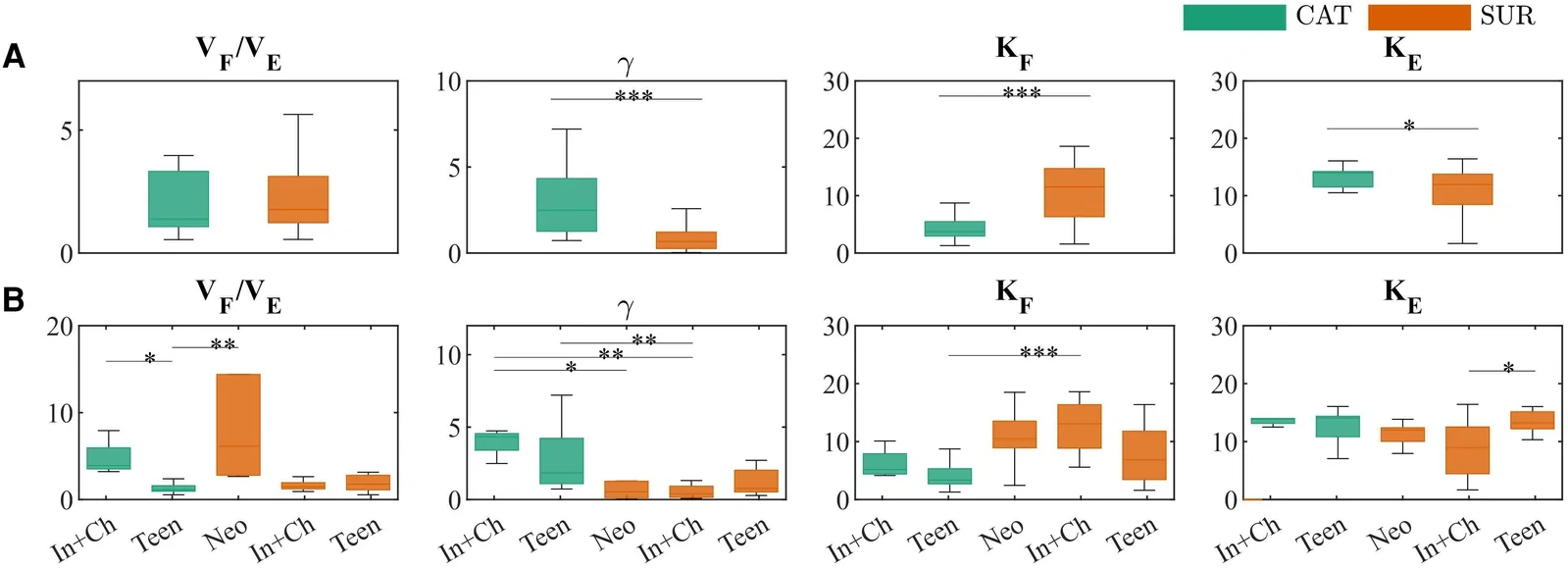
Estimated model parameters grouped by age and intervention. Distributions of model parameter values representative of physiological processes controlling glucocorticoid dynamics in pediatric patients. The parameter value distributions were grouped by catheter (CAT) and surgery (SUR) interventions (A), as well as by age group (B). V_F_ = velocity of cortisol to cortisone conversion, V_E_ = velocity of cortisone to cortisol conversion, K_F_ = Michaelis–Menten constant for cortisol to cortisone conversion, K_E_ = Michaelis–Menten constant for cortisone to cortisol conversion, g = rate of hormone removal from the system. Neo = Neonates, In + Ch = Infants + Children, Teen = Teenager.

Figure 5 illustrates the estimated distributions of parameter values by type of intervention (Fig. 5A) and across age groups (Fig. 5B). The ratio V_F_/V_E_ quantifies the balance between the velocity of cortisol to cortisone conversion (V_F_) and the velocity of cortisone to cortisol conversion (V_E_), thus serving as an indicator of the directionality of the dynamic interconversion between these hormones (Fig. 4). The V_F_/V_E_ ratio was not different between the catheter and surgery groups but was considerably higher in neonates than in any other age group. The rate of removal from the system (g) was also lower in the surgical group, regardless of age. The surgical group also had higher K_F_ values (inversely proportional to half the maximum rate of cortisol to cortisone conversion) than the catheter group across all ages. The surgical group had slightly lower K_E_ values than the catheter group, but they were broadly similar across age groups.

## Discussion

The role of the HPA axis, especially cortisol, in neonates and children undergoing cardiac surgery is still controversial. Clinical trials, translating into many systematic reviews (4, 23), often conflate 2 questions: is there a group deficient in corticosteroids at surgery, and does supranormal corticosteroid administration improve outcomes?

Neonates had lower blood values of total cortisol and CBG than other age groups during and after cardiac surgery, but higher peak values and AUC of free cortisol and cortisone in their interstitial fluid than other age groups during and after the surgery. The discrepancy between total and free cortisol in neonates is likely due to lower CBG concentrations in neonates, consistent with previous work (24). Lower levels of CBG mean that small changes in blood total cortisol translate into large, exaggerated changes in the free, active fraction of cortisol available to diffuse into the tissues. Adult levels of CBG are not reached until 6 months after birth (25, 26).

Neonates undergoing surgery had higher cortisone levels in microdialysis samples than those in other age groups. The data on older children are not discussed in detail herein because no clear conclusions could be drawn and because the neonates emerge as a distinct group. Our mathematical modeling suggests this is due to a higher cortisol-to-cortisone conversion rate by 11β-HSD2 than a cortisone-to-cortisol conversion rate by 11β-HSD1 (Fig. 4). The estimated Michaelis–Menten constant (K_F_) in neonates was similar to older age groups, implying greater 11β-HSD2 activity rather than affinity and suggesting the persistence of fetal and peripartum 11β-HSD2 activity over lower 11β-HSD1 activity converting high concentrations of free cortisol (27). Cortisol concentrations rise, and cortisone concentrations fall until reaching near adult values over the first year of life (28)—although this pattern continues throughout life (29). This first year also entrains the circadian rhythm, with high morning serum cortisol concentrations at 1 year after birth (27).

Consistent with previous findings (30, 31), the hormone profiles of pediatric patients show ACTH as the primary driver of cortisol changes, with ACTH peaking before cortisol. Inflammatory mediators are highest in neonates and affect cortisol concentrations in vivo (32) and in vitro (33). Cortisol remains under pituitary control in all children undergoing surgery, despite the previously reported pituitary-adrenal gland disconnect (33). High-frequency microdialysis allows measuring systemic dynamic changes, unlike single-time point blood tests that may show reduced ACTH values. Static tests miss the ACTH-cortisol interaction observed in children's heart surgery, as in adult heart surgery (11) and critically ill patients (34), albeit with altered sensitivity.

The strength of this study is in its high sampling frequency, quantifying the HPA axis dynamic response to cardiac surgery and enabling calibration of a mathematical model to understand HPA axis complexity in vivo. Simultaneous blood and interstitial fluid sampling provides insights into the physiology of cortisol metabolism in children. This is important because it is the combination of the size and frequency of the signals that ultimately determines HPA axis dynamics. Feedforward signals from ACTH, cortisol feedback, inflammatory mediators and indirect signals such as splanchnic nerve tone, CBG concentrations and metabolism all govern peripheral cortisol concentration simultaneously (35). This complexity cannot be modeled from in vitro samples or single time-point blood samples. Despite the small number of participants, there is a high number of samples per participant. This high-density data sets is what facilitates the application of nonconventional approaches such as statistical time series analysis and mathematical modeling, and consequently the identification of digital biomarkers that distinguish between subgroups. Another strength of this study lies in recruiting patients across the pediatric cardiac surgery age range, allowing for observation of HPA axis changes throughout childhood.

Some limitations exist. We recruited a few patients in specific age groups, preventing subgroup analyses on congenital defect, procedural complexity, or puberty's impact on cortisol response. We did not recruit patients aged 6-10 for surgery or the catheter suite, so we cannot comment on this age group. This age group has a low frequency of cardiac intervention as many lesions are corrected or palliated in the first 2 years after birth and do not usually require further intervention until puberty. Some age differences observed in the hormonal patterns may be due to surgical insult or cardiac lesion severity not the endogenous HPA axis response of age. Serum data were less frequent than microdialysis, so we could not include them in the mathematical model. While the model accurately predicted cortisol and cortisone levels in many patients, it was not accurate for all (Fig. S4). This highlights that our model predicts population values, not individual patients. Whilst the model performed marginally better for the catheterism group than the surgical one, the high coefficient of determination R^2^ found in both cohorts further attests to the validity of the underlying assumptions.

The aggregated data align with previous findings from single-time-point sampling of HPA axis hormones: patients undergoing cardiac surgery have higher cortisol and inflammatory mediator concentrations than those undergoing catheter procedures (36). Many of the results also match previous studies (eg, low CBG in neonates) (24). This consistency suggests our approach for quantitative characterization and mathematical modeling is accurate.

## Conclusion

Children undergoing cardiac surgery exhibit higher cortisol concentrations compared with those undergoing procedures in the cardiac catheterization suite. Neonates differ physiologically from older children. They have lower total cortisol levels, but a greater proportion of free (unbound) cortisol, which more readily diffuses into interstitial tissues. As a result, neonatal tissue cortisol levels may actually be higher. These physiological differences mean that thresholds used to define cortisol deficiency in older children cannot be directly applied to neonates in the perioperative setting. Accordingly, neonates require dedicated investigation into HPA axis pathophysiology, as well as tailored approaches to diagnosis and management. Thresholds for both deficiency and supplementation may differ significantly from those established in older pediatric populations.

## Data Availability

The data that support the findings of this study are not publicly available but are available from the corresponding author upon reasonable request and with appropriate approvals.
